# Nuclear Imaging in Pediatric Cardiology: Principles and Applications

**DOI:** 10.3389/fped.2022.909994

**Published:** 2022-07-06

**Authors:** Maelys Venet, Mark K. Friedberg, Luc Mertens, Jerome Baranger, Zakaria Jalal, Ghoufrane Tlili, Olivier Villemain

**Affiliations:** ^1^Division of Cardiology, Department of Pediatrics, The Hospital for Sick Children, University of Toronto, Toronto, ON, Canada; ^2^Department of Congenital and Pediatric Cardiology, Hôpital du Haut-Lévêque, CHU de Bordeaux, Bordeaux-Pessac, France; ^3^Department of Nuclear Medicine, Hôpital du Haut-Lévêque, CHU de Bordeaux, Bordeaux-Pessac, France

**Keywords:** pediatric cardiology, nuclear imaging, radiopharmaceutical, scintigraphy, positron emission tomography

## Abstract

Nuclear imaging plays a unique role within diagnostic imaging since it focuses on cellular and molecular processes. Using different radiotracers and detection techniques such as the single photon emission scintigraphy or the positron emission tomography, specific parameters can be assessed: myocardial perfusion and viability, pulmonary perfusion, ventricular function, flow and shunt quantification, and detection of inflammatory processes. In pediatric and congenital cardiology, nuclear imaging can add complementary information compared to other imaging modalities such as echocardiography or magnetic resonance imaging. In this state-of-the-art paper, we appraise the different techniques in pediatric nuclear imaging, evaluate their advantages and disadvantages, and discuss the current clinical applications.

## Introduction

Pediatric cardiology includes heterogeneous congenital and acquired heart diseases. Echocardiography is the key non-invasive diagnostic imaging tool that is usually sufficient to manage most children with heart disease (1, 2). However, cross-sectional imaging modalities such as magnetic resonance imaging (MRI) and computed tomography (CT) are increasingly used in pediatric cardiology, and can provide additional anatomical, structural, and functional information (3–5). In adult cardiology, nuclear imaging techniques are used for evaluating myocardial perfusion and viability, pulmonary perfusion, ventricular function, and detection of inflammatory processes. The role of nuclear imaging is more controversial and less well established in pediatric cardiology. The evolution of scanning technologies and the development of low radiation dose protocols dedicated to children open new perspectives on the use of nuclear imaging in pediatric heart disease. This state-of-the-art paper aims to highlight the principles and the current roles of nuclear imaging in pediatric cardiology and discuss its current limitations.

## Technical Considerations

### Functional and Molecular Imaging: Basic Principles

The aim of nuclear imaging is to visualize, characterize and quantify biological processes by non-invasive methods. The imaging contrast is provided by radiopharmaceuticals that behave as biological probes. The pharmaceutical is combined with a radionuclide, used for detection, and their biological properties determine the diagnostic significance of the radioactive signal (6). The main advantage of this technique relies in the ability to quantify, map, and monitor a specific biological activity *in situ*, with high sensitivity and specificity (7). It can represent a specific metabolic activity, receptor density or blood flow (8). Due to the inherent characteristics of the radionuclide signal and its detection mode, the spatial resolution of nuclear imaging is lower than that of other clinical imaging techniques (2.5 –5 mm for PET, 5–10 mm for SPECT versus 0.5–1 mm for MRI or CT) (9).

### Scintigraphy, Single-Photon Emission Computed Tomography, Positron Emission Tomography, and Hybrid Systems: From Physics to Practice

There are three main types of nuclear imaging techniques that differ in their detection method: (a) two-dimensional (2D) gamma scintigraphy, (b) single-photon emission computed tomography (SPECT) and (c) positron emission tomography (PET). Scintigraphy and SPECT is to detect gamma radiation from photon emitting radiotracers, using gamma ray detectors. While planar scintigraphy is a 2D detection method, providing a projection image like a classic X-ray, SPECT allows acquisitions in the three dimensions (3D). Multiple 2D images from several angles around the patient are acquired and computed tomographic reconstruction algorithm is used to generate a 3D data set (6). In the same way as SPECT, PET reconstructs volumetric acquisitions from of a set of 2D images, but the detection method and the radionuclides used are different. PET is based on indirect detection of positrons since the PET camera detects the annihilation photons created by the positron-electron interaction in the biological environment. Currently, most PET cameras integrate an X-ray computed tomography (CT) scanner (10). Tomographic reconstruction must implement corrective measures to avoid artifacts generated by interactions between photons and matter (11). In case of hybrid PET/CT and SPECT/CT cameras, this process is based on CT transmission scanning, which provide a map of attenuation coefficients (12). Hybrid systems provide, in a single imaging study, comprehensive cardiac assessment consisting of accurate anatomic mapping and artifact-free molecular information (10). However, PET and CT images are not acquired simultaneously, and potential misalignment, due to respiratory motion for example, need to be considered during the PET/CT fusion images reviewing.

### Radiopharmaceuticals: Concept of Molecular Targeting

Radiopharmaceuticals consists of three components: a vector molecule, a radionuclide, and a linker in between. They are administered at a nano-molar concentration and accumulate in the targeted tissue based on the specific molecular vector-target interaction. The radiotracer is most often administrated intravenously but inhalation [e.g., ventilation scintigraphy with inhaled radioactive gas (13)] or ingestion [e.g., orally administered fluorine-18 fluorodeoxyglucose (18F-FDG) (14)] are also used. The radioactive label is used diagnostically as an emitter of electromagnetic radiation. The vector molecule can consist of a small molecule, a peptide, a protein including antibodies or a nanoparticle (15). Vectors and radionuclide have different properties and therefore lead to different applications (Table 1). The choice of a radiopharmaceutical is impacted by its biological and nuclear characteristics, its availability, and its cost which depend on the way it is manufactured (16). Their production typically requires a nuclear reactor or medical cyclotron. The nuclear reactors allow a large-scale production of most of radioisotopes at lower cost by bombarding a target with neutrons that cause fission reactions. The cyclotrons are circular particle accelerators which are usually installed in large hospitals and produce, in a more limited and expensive way, only some radioisotopes as Fluor-18 and Thallium-201 for an immediate use. Radionuclide generators can also be used as a more convenient and portable source to provide some selected radiopharmaceuticals (17–19). The most common is the technetium-99m generator, a space-saving device stored in the hospital radiopharmacies and used to extract technetium-99m from a molybdenum-99 source, for a direct use in nuclear medicine diagnostic procedures.

**TABLE 1:** The main radiopharmaceuticals in pediatric cardiology.

	Radionuclide production	Physical half-life	Biological properties	Indications	Refs.
**Single photon emitters**					
99mTc-tetrofosmin	Generator	6 h	Lipophilic cationic agent: passive myocardial uptake proportional to the regional MBF	MPI; Ventricular function	(19)
Tl-201	Cyclotron	73 h	K + analog: active myocardial uptake proportional to the regional MBF	MPI; MBF quantification; Viability	(74)
99mTc-MAA	Generator	6 h	Human albumin aggregates: trapped in capillaries	LPS; Right-to-left shunts	(19)
123I-MIBG	Cyclotron	13 h	NE analog: sympathetic innervation marker	Autonomic imaging	(18)
**Positron emitters**					
18F-FDG	Cyclotron	110 min	Glucose analog: marker of high cellular/tissular glucose uptake	Infection; Malignancy; Viability	(15)
Rubidium-82	Generator	75 s	K + analog: active myocardial uptake proportional to the regional MBF	MPI; MBF quantification; Viability	(15, 19)

*FDG, fluorodeoxyglucose; LPS, lung perfusion scintigraphy; MAA, macroaggregated albumin; MBF, myocardial blood flow; MIBG, meta-iodobenzylguanidine; MPI, myocardial perfusion imaging; NE, norepinephrine.*

## Clinical Applications

### Myocardial Perfusion

#### Principles and Protocols

**FIGURE 1:**
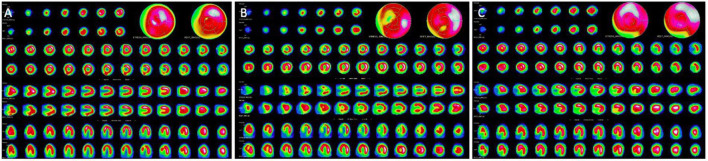
Overview of nuclear imaging in pediatric cardiology. CT, computed tomography; PET, positron emission tomography; SPECT, single-photon emission computed tomography.

**FIGURE 2:**
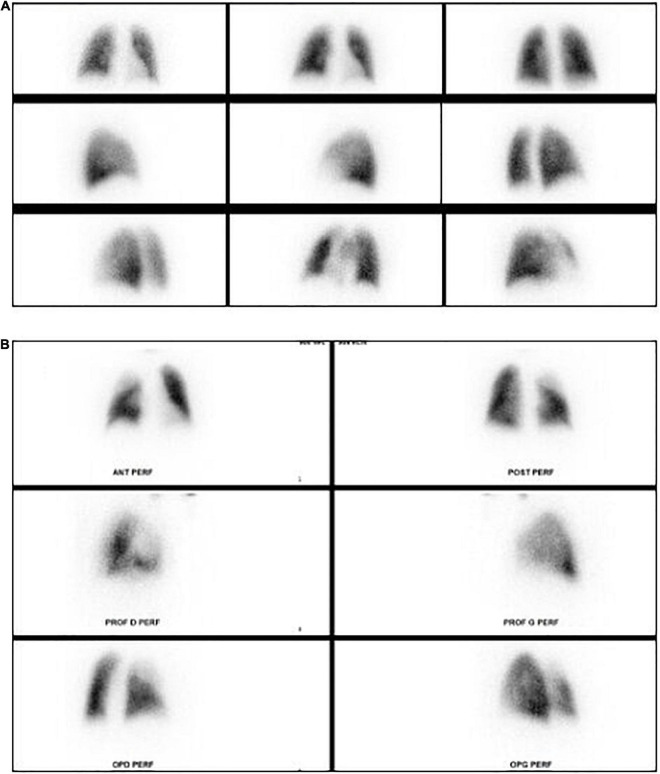
Stress and rest myocardial perfusion SPECT. **(A)** Normal stress and rest myocardial perfusion SPECT. **(B)** Stress-induced antero-septal and antero-apical ischemia in the setting of left anterior descending artery critical stenosis. **(C)** Fixed inferior perfusion defect on both rest and stress acquisitions: this pattern can be related to scarring form infarction or hibernating myocardium.

Nuclear myocardial perfusion imaging (NMPI) provides both metabolic and functional analysis (Figure 1). The commonly used radiopharmaceutical agents for NMPI in children, as in adults, are SPECT radiotracers: thallium-201 chloride (Tl-201) and technetium-99m-labeled agents (20, 21). After intravenous injection of the radiotracer, its myocardial uptake is proportional to the regional myocardial blood flow (MBF) (9). When coronary perfusion is impaired, the uptake of the radiotracer is decreased proportionally to the regional flow (Figure 2). When MBF is preserved at rest, exercise or pharmacological stress (injection of dipyridamole, dobutamine, adenosine or selective adenosine receptor agonists such as regadenoson) can unmask underlying ischemia.

Myocardial perfusion and viability can also be assessed by PET/CT. Myocardial viability is identified as a mismatch between fluorine-18 fluorodeoxyglucose (18F-FDG) uptake and rest perfusion imaging. A viable myocardial segment is defined by reduced or absent perfusion with preserved glucose metabolism (22). PET agents, such as Tl-201 SPECT, can provide an absolute quantification of the MBF and determine the myocardial flow reserve (23).

#### Congenital Coronary Abnormalities

Congenital coronary abnormalities are a clinical challenge both diagnostically and therapeutically with a lot of controversies regarding the related individual risk and the management. Anomalous aortic origin of a coronary artery from the opposite sinus of Valsalva (AAOCA), associated with intramural or interarterial course, is increasingly diagnosed incidentally in children, but the related clinical risks are not well defined (24). A key component in the assessment of these patients include evaluation of regional myocardial perfusion at rest and during physical or pharmacological stress. The current guidelines from the American Heart Association/American College of Cardiology are based on anatomic findings, the presence of symptoms and the results of the stress testing to guide exercise limitations (25). However, the recent guidelines from the American Society of Echocardiography also emphasize the as yet unclear role of NMPI in management of asymptomatic patients with AAOCA (26). The non-irradiating alternative technique to assess for inducible myocardial hypoperfusion, wall motion and myocardial viability, is the dobutamine stress-cardiac MRI. It can be performed safely in pediatric patients with AAOCA and has shown promising results in risk stratification and decision-making in this challenging patients (27).

The use of NMPI has been favorably reported in other rare congenital coronary disease such as left coronary artery originating from the pulmonary artery (ALCAPA) (28, 29), myocardial bridging (30), Williams syndrome with coronary involvement (31) or complex CHD such as pulmonary atresia-intact ventricular septum with right ventricular coronary sinusoids (32). In ALCAPA, identification of hibernating myocardium is useful to evaluate the chances of recovery after surgical repair. It can be demonstrated by nuclear imaging as a mismatch between reduced rest perfusion and enhanced glucose uptake on PET imaging (33).

#### Transposition of Great Arteries Corrected by Arterial Switch Operation

Both early and late mortality after arterial switch operation (ASO) for transposition of great arteries (TGA) is predominantly related to coronary artery complications (34–36). Exercise testing with ECG alone has been shown to be insufficient in identifying ischemia after ASO (37, 38). Perfusion defects diagnosed by NMPI are present in 5–24% of patients during follow-up and persist more than 10 years after ASO (39–41). Analyzing 110 children with TGA after ASO, Sugiyama et al. directly compared angiographic and NMPI findings and concluded that SPECT is accurate to differentiate spontaneously resolutive from progressive stenoses in this population (42). In addition, the authors propose that the indication for coronary reintervention should not depend on angiographic findings alone, but also on evidence for myocardial ischemia. In that study, significant angiographic lesions were not always related to an evolving stenotic process, as some resolved over time. For the angiographic lesions that normalized over time, the initial SPECT was normal while it was always abnormal for the progressive lesions. Similar studies show the usefulness of NMPI (SPECT or PET) in TGA/ASO adolescents with an impact on decision-making (39, 43, 44). However, the performance of a perfusion test seems relevant only in symptomatic patients or in those with a postoperative history of myocardial ischemia and should be combined with anatomic imaging (MRI, CT or angiography) (40, 41, 45).

#### Kawasaki Disease

Kawasaki disease (KD) is an acute pediatric vasculitis of unknown etiology that results in coronary artery aneurysms in up to 25% of untreated cases (46) and 5% of appropriately treated cases (47). Coronary stenoses may also develop during the healing phase or late after the acute episode (48). The utility and safety of NMPI as a non-invasive monitoring modality for coronary stenosis progression in KD has been demonstrated (49, 50). The most recent North American and Japanese KD guidelines discuss the use of NMPI in the long-term follow-up. They recommend that inducible myocardial ischemia testing (stress testing) is indicated every 1–5 years in patients who have or had coronary aneurysms (including small and/or resolved aneurysms) or in patients with symptoms or ventricular dysfunction (Class IIa; Level of Evidence B) (46, 51). However, these guidelines do not recommend when to perform stress echocardiography versus stress MRI or stress nuclear imaging.

In children with a history of KD, 99mTc SPECT provides a sensitivity of 90% and a specificity between 85 and 100% in detecting stress-induced ischemia related to significant coronary stenosis (52, 53) and allows to monitor the worsening or improvement of angiographic stenoses over time (50). The sensitivity of the 201Tl SPECT for this indication is equivalent (54).

In KD, regardless of a history of aneurysms, 12–19% of patients have an abnormal NPMI pattern with permanent and/or stress-induced perfusion defects during the follow-up (55–57). Interestingly, in over the 370 patients included in three different studies (55–57), there was the same proportion of perfusion defects in patients with and without history of coronary aneurysms. The authors propose that these findings may be related to microcirculatory damage occurring during KD, independent of involvement of the larger coronary arteries. In adolescents with a history of KD during infancy, N-13 ammonia perfusion PET demonstrated a decrease in coronary flow reserve after adenosine-induced vasodilatation compared with controls, implying the presence of long-term coronary endothelial dysfunction (58). These data show the ability of NMPI techniques to study the coronary physiology at the microvascular level and the importance of the long-term follow-up and cardiovascular secondary prevention in all patients with a history of KD.

#### Cardiomyopathies

In hypertrophic cardiomyopathy (HCM), myocardial ischemia has been suggested to contribute to the pathophysiology of the disease, and appears to be related to decreased subendocardial perfusion in the hypertrophied segments, compression of intramural small vessels and myocardial bridging (59). Microvascular ischemia is thought to be involved in the development of adverse ventricular remodeling, and diastolic and systolic dysfunction, impacting clinical outcomes in adults and children (60–64). NMPI can contribute as a reliable non-invasive methods for the detection of myocardial ischemia, adding to risk stratification and treatment (59, 62). However, the current guidelines suggests that stress echocardiography should be preferred in these patients since it also allows to diagnose LV outflow tract obstruction which is a common concern in this population (65).

In pediatric dilated cardiomyopathies, NMPI can rarely be useful to non-invasively rule out underlying ischemic process, which is an uncommon etiology in children. For pediatric diseases affecting coronary microvascular perfusion such as sickle cell anemia with LV dilatation and/or dysfunction, it can help to differentiate between the possible mechanisms causing left ventricular damage (66). In these patients, microcirculatory abnormalities can be a rare cause of atrioventricular block, which can be demonstrated by NMPI despite normal angiography (67).

#### Heart Transplantation

One of the long-term complications of heart transplantation (HT) is the development of cardiac allograft vasculopathy (CAV). In children, CAV is a major cause of death and retransplantation (68, 69). The disease involves both distal and proximal coronary arteries and is associated with functional anomalies such as systolic dysfunction and increased filling pressures (69). Coronary arteriography is the recommended technique for follow up in adult and pediatric HT recipients. However, CAV diagnosis is challenging, even with angiography, due to the involvement of the distal vasculature and microvascular changes (70). Indeed, Maiers et al. showed that the most accurate strategy to diagnose CAV was through a multimodality approach combining echocardiography, myocardial perfusion assessment including stress SPECT and coronary angiography (71).

### Lung Perfusion

Lung perfusion scintigraphy (LPS) performed with 99mTc labeled macroaggregated albumin (MAA) is the most widely used nuclear imaging technique to quantify lung perfusion and relative flow distribution (72, 73). The labeled particles are distributed according to the pulmonary flow into both lungs and the results are expressed as a percentage of total pulmonary flow for each lung (13). LPS is still considered as the clinical reference technique for the evaluation of pulmonary blood flow distribution (74–76) and has been used in children with CHD for over 50 years, at any age (77). Quantitative LPS is based on multiple planar acquisitions (at least anterior and posterior projections) with no specific preparation and no need for sedation, even in infants (75). Instead of planar acquisitions, SPECT can be used to obtain 3D imaging of the pulmonary perfusion and is recommended by some investigators who highlight the better image contrast (higher sensitivity) for equivalent safety (78, 79). SPECT however can require sedation in younger children to ensure immobility of about 15 min versus 2 min needed for planar LPS (74).

**FIGURE 3:**
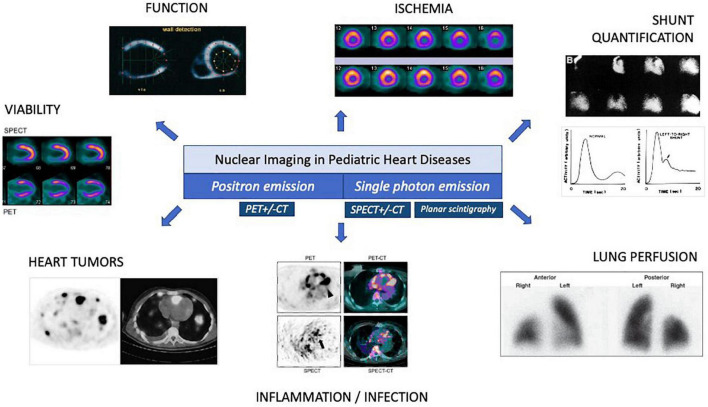
Lung perfusion planar scintigraphy. **(A)** Normal lung perfusion scintigraphy. **(B)** Pulmonary perfusion defect in the right upper lobe in patient with pulmonary embolism.

The main indication for LPS is the assessment of hemodynamic impact of pulmonary vascular abnormalities including pulmonary artery or vein stenosis, pulmonary emboli (Figure 3), arterio-venous fistula, and aorto-pulmonary collaterals (13, 80–82). In pulmonary arteries stenosis, LPS provides key information about the hemodynamic impact of the stenosis. The North American guidelines for pediatric cardiac catherization defined as significant stenosis when there is relative flow discrepancy between the 2 lungs of 35%/65% or worse (83). LPS also is one of the imaging techniques that can be used for etiological assessment of pulmonary arterial hypertension (PAH) in children (84, 85). In pulmonary vein stenosis, the LPS sensitivity is 72% and specificity is 83% for all veins when compared with the angiographic findings (86). When compared to phase contrast MRI, LPS has advantages and limitations with similar diagnostic accuracy when performed by trained teams (87). The major benefit of LPS is the simple and fast execution without the need for sedation at any age. Interpretation errors, often related to confounding factors impacting pulmonary flow, can be avoided by careful choice of the injection site (i.e., preferential caval flow to one lung in Fontan circulation) and by knowing the surgical history (74, 88). A rare but potential remaining pitfall is the symmetrical bilateral pulmonary artery stenosis which can result in symmetrical relative perfusion to both lungs (75). Lastly, the major intrinsic limitation of LPS is the ionizing radiation. The current doses are low [0.5–2 MBq/kg (13)] but MRI can be preferred to avoid this radiation exposure in children (87, 89).

### Inflammation and Infection

#### Endocarditis

##### 18F-FDG PET/CT

Nuclear imaging and more specifically 18F-fluorodeoxyglucose (18F-FDG) PET allows visualization of inflammatory processes through the mapping of cellular glucose uptake. The 18F-FDG is a glucose analog in which the carbon-2 hydroxyl is replaced by fluorine-18, a radioisotope that decays to oxygen-18 by positron emission. The heart metabolizes both carbohydrates and free fatty acids and absorbs a large amount of 18F-FDG. A preparatory low carbohydrate diet and a fasting period are required to suppress the physiologic myocardial 18F-FDG uptake, making free fatty acids the predominant cardiac energy source and improving the contrast effect (90). 18F-FDG PET/CT is used in children to interrogate various malignancies, based on the high glycolytic activity in malignant cells (91). Inflammatory imaging also has relevant applications in pediatric cardiology, particularly to diagnose infective endocarditis (IE), vasculitis and heart tumors.

Overall IE incidence in CHD patients is 1.33 per 1,000 person-years with a high proportion of right-sided IE (92, 93). Prosthetic valves derived from bovine jugular veins are one of the most risky substrates for IE (94). Pacing device and ventricular assist device infections are also commonly represented in this population (95). The latest European and North American guidelines emphasize the role of the 18F-FDG PET/CT in prosthetic valve endocarditis (PVE) diagnosis (96, 97). However, it is not recommended in the work-up of native valve endocarditis due to poor sensitivity in this setting (98). Although these recommendations were based on studies including left heart valve prostheses, recent works has validated the performance of PET/CT for right heart prostheses and conduits, implanted pacing devices and VADs in children and adults (99–104). As for all nuclear imaging techniques, there is no age limit to perform a cardiac 18F-FDG PET/CT for suspected PVE, with the youngest patient described in the literature being 1 year old (105). So far, no study has evaluated 18F-FDG PET/CT imaging for diagnosis of PVE in a specific pediatric population but there is no reason to believe that the performance would differ in the pediatric subgroup, when the appropriate protocols can be applied (99).

The addition of the PET/CT to the Duke criteria substantially increases the diagnostic yield of this score (sensitivity from 70 to 97%) (96) with a special interest in conditions where the sensitivity of echocardiography is typically low, such as infections of pulmonary outflow prostheses, VAD or intracardiac leads, where the presence of vegetations is relatively uncommon or difficult to image (95, 99, 106). In addition, PET/CT can diagnose septic embolization with higher sensitivity than CT and can also help to identify culprit lesion (99, 107). The main limitations of cardiac PET/CT in children are its availability, the need for preparatory fasting and the radiation dose [up to 25 mSV for a whole-body PET/CT in children (108)]. However, radiation dose has decreased considerably in recent years with recent PET/CT technologies, such as digital PET/CT, allowing to decrease the injected activity of the radiopharmaceutical (109).

##### Radiolabeled Leukocytes Scintigraphy

Amongst the nuclear imaging techniques used in diagnosis of infectious diseases, radiolabeled leukocyte scintigraphy (RLS) is technically more demanding but also more specific for bacterial infection than 18-F FDG PET/CT (110, 111). Although not widely used in clinical practice, its performance in the diagnosis of infections of unknown origin in children has been recognized, also in neonates (112, 113). This technique, however, is based on a complex procedure including manual cellular marking of the patients’ leukocytes, either using 111In-oxine or 99mTc hexamethylpropyleneamine oxime (HMPAO), and repeated acquisitions over 2 days with a long imaging duration (114, 115). Leukocyte turnover is slower in infectious processes, which distinguishes it from inflammation. Typically, the diagnosis of infection is made when there is a stable or enhancing focus of leukocyte accumulation between the early (4–6-h) and delayed (18–24-h) images (114). RLS has been included in the most recent modified Duke criteria as a major criterion for the PVE diagnosis (96).

#### Cardiac Malignancy

Although nuclear imaging is widely used in pediatric oncology, its use in the field of pediatric cardiac tumors is still very limited. Pediatric cardiac tumors are benign in 90% of cases and the diagnosis is largely based on echocardiography and/or MRI (116, 117). In case of atypical presentation and concern for malignancy, 18F-FDG PET/CT could potentially help to differentiate between benign and malignant processes. It can also identify the primary lesion in metastatic disease and is of use in staging the hematological malignancies. It can help to select biopsy location or even guide radiotherapy (118). Data on the use of 18F-FDG PET/CT in pediatric cardiac tumors are, however, missing. A few cases of identification of myocardial metastases based on whole body 18F-FDG PET/CT have been reported (119, 120) but no study has specifically investigated the role of nuclear imaging in diagnosis of pediatric cardiac tumors.

### Assessing Ventricular Systolic Function

Multigated acquisition (MUGA) of the cardiac blood pool, also called equilibrium radionuclide ventriculography, is the traditional nuclear imaging modality to assess ventricular systolic function to quantify ventricular ejection fraction. This method requires the labeling of the patient’s red blood cells with a radionuclide (usually 99mTc), which can be done *in vivo* or *in vitro*. The cardiac chambers are then visualized based on the radioactive emission and the LV volume is measured over the cardiac cycle to assess the ejection fraction. This technique was first validated in adults in the early 1970s (121) but also performed in children with CHD for decades (122–124). Systolic function can also be assessed by contouring the myocardial wall and not the chamber as in ventriculography. This second technique is used to evaluate LV ejection fraction during NMPI examinations, using the same radiopharmaceutical injection (45, 66).

Finally, even though radionuclide ventriculography is available and reliable in children, there is no relevant indication given the existence of non-irradiating techniques such as echocardiography and MRI which also allow a more precise study of the cardiac function.

### Shunts Quantification

#### Left-to-Right Shunts

The aortic (Qs) to pulmonary (Qp) flow ratio (Qp/Qs) is a key parameter in the management of patients with intracardiac shunts in pediatric cardiology. Nuclear imaging can be useful to directly measure shunt flow and provide a non-invasive Qp/Qs assessment. The technique involves the rapid injection of a compact bolus of radionuclide (usually 99mTc agents) while monitoring the transit through the heart and lungs with the gamma camera. Planar acquisitions are performed with a high sampling rate and dedicated collimator to magnify the images. For quantification, the time-radioactivity curves are generated from regions of interest: superior vena cava to assess the quality of the bolus and periphery of the right, left or both lungs (pulmonary curve) for shunt detection and quantification (125). The normal pulmonary curve has a first large peak related to the first pass of the bolus to the lungs and a second smaller peak which represents the physiological recirculation. In left-to-right shunts, the second peak is much earlier, creating a shoulder in the downslope of the first peak because of abnormal early recirculation through the shunt (126). The quantification of the Qp/Qs is computer-assisted, based on the curve fitting method and a gamma variate extrapolation (125).

While it remains a simple and reliable way to non-invasively quantify the Qp/Qs, its use has become obsolete in current clinical practice as non-radiation based techniques have largely replaced nuclear methods for shunt assessment (127, 128).

#### Right-to-Left Shunts

Using the same principle as described above, right-to-left shunts can be detected by first-pass method, which reveals a premature appearance of radioactivity in the left chambers. The time-radioactivity curves are generated from regions of interest over the carotid artery to quantify the shunt impact (125). The most widely used radiopharmaceutical is 99mTc-labeled macroaggregated albumin particles, such as those used for the assessment of pulmonary perfusion. These particles are physiologically trapped in the lungs but in case of right-to-left shunt, they are distributed over the systemic flow and retained in the systemic capillary networks relative to the Qp/Qs ratio. The magnitude of the shunt is obtained by comparing lung with brain counts or with total extrapulmonary activity on a whole-body image (73). While reliably technique, it has been largely replaced by other imaging tools.

### Autonomic Imaging

Postoperative alteration of the cardiac autonomic innervation is frequently described after cardiac surgery and is likely due to direct damage of the cardiac nervous system. This can also be caused by prolonged aortic cross-clamp and coronary manipulations leading to autonomic fiber ischemia (129, 130). Heart transplantation results in complete cardiac denervation with clinical manifestations including higher heart rate, increased blood pressure and systemic vascular resistance with impaired orthostatic responses (131). Nuclear imaging allows investigation of the abnormal cardiac autonomic innervation by using radiotracers analogs of norepinephrine. Different studies have been conducted to investigate the correlation between abnormal cardiac norepinephrine uptake and clinical events, both in adults (132) and children (133). Interestingly, in ischemic cardiomyopathies, autonomic imaging may better predict life-threatening ventricular arrythmias than parameters as LVEF (132). Some teams have also shown that the heterogeneity of meta-iodobenzylguanidine (MIBG) uptake was correlated with risk of arrhythmia in patients with tetralogy of Fallot (134) or in congenital long QT syndrome (135). In another hand, Possner et al. have shown that the incomplete cardiac reinnervation late after the arterial switch operation for TGA can at least partially explain the reduced myocardial perfusion response to sympathetic pharmacological stimulation in these patients (130). However, the prognosis and the clinical impact of the pathological patterns of norepinephrine cardiac uptake remain incompletely understood and autonomic nuclear imaging is not routinely performed in cardiology and pediatric cardiology.

Cardiac autonomic imaging has also been used in vasovagal syncope work-up as exaggerated MIBG LV uptake is considered as a good marker of sympathetic hyperactivity in children with suspected neurocardiogenic syncope (136). Although it is rarely used in practice to manage these patients, these data support the benefit of cardioselective β-blocking agents in the therapy of recurrent syncope in children.

## Limitations

Each nuclear imaging technique has its advantages and limitations, in technique or application, as detailed in previous sections. The main common limitation is the risk related to ionizing radiation and its long-term impact. The underlying neoplastic risk appears particularly important in children due to the increased radiosensitivity of this population with higher mitotic activity and longer potential for exposure over the life span (137). This risk is higher in children with chronic diseases, such as congenital heart disease (CHD), who undergo long hospital stays with multiple diagnostic or therapeutic irradiating procedures (138).

To quantify and compare the doses of ionizing radiation induced by nuclear imaging, it is necessary to express them in equivalent dose. This parameter, expressed in mSv, depends on the nature of the radiation and the absorbed dose (the energy received per unit of mass). Finally, the effective dose is the equivalent dose corrected by a tissue weighting factor to consider the specific sensitivities of the different irradiated organs. This effective dose is calculated for the whole body and used to assess the biological risk related to the radiation exposure. Effective doses therefore vary widely depending on the nuclear imaging techniques, the radiopharmaceutical used and the patient’s characteristics.

There are continuing efforts to determine, standardize, and reduce ionizing radiation exposure and dosage in pediatric nuclear imaging protocols (139–142). For most pediatric studies, radiopharmaceutical optimal doses are based on an adult references activity according to the patient’s weight and/or the body surface area (BSA) and pediatric-specific guidelines are available (141–143).

Over the last decade, significant decreases in radiation exposure has been made possible by the technological advances in camera sensitivity and image processing (144, 145) and more accurate risk-benefit assessment through computational absorbed-dose models (146). The development of hybrid imaging combining nuclear imaging and MRI is another important step in reducing the CT component of the total radiation dose (147–150).

An additional limitation of nuclear imaging in pediatrics is its accessibility. Depending on the radiotracers used, the availability of a cyclotron in the hospital or the hospital’s proximity to a nuclear reactor can be essential and makes certain techniques available in only a few centers.

## Conclusion

Following technical improvements in ultrasound and the emergence of additional imaging techniques such as 4D flow MRI or stress MRI, nuclear imaging is now a second- or third-line modality for many indications in pediatric cardiology. According to the literature review, the main current indications for nuclear imaging in children are (Table 2): 1/myocardial perfusion and viability imaging in the setting of congenital or acquired coronary abnormalities, 2/lung perfusion imaging to quantify the impact of pulmonary arteries stenosis and indicate intervention, 3/inflammation imaging in the setting of prosthetic valve or device-related endocarditis when the diagnosis is uncertain by other techniques. As nuclear imaging can provide unique information on cellular metabolism, molecular processes, and/or blood flow distribution, it should be part of the armamentarium of available imaging modalities for clinical assessment in pediatric cardiology.

**TABLE 2:** Characteristics of most relevant nuclear imaging applications in pediatric cardiology.

	Myocardial perfusion		Myocardial viability		Lung perfusion	Inflammation imaging
Main indication	Congenital or acquired coronary abnormalities				Pulmonary arteries stenosis	Material-related endocarditis
Technique	SPECT	PET	SPECT	PET	Planar scintigraphy	PET
Radioisotopes	Tc-99m agents Tl-201	Rb-82 N13-NH3	Tl-201	18F-FDG Rb-82	99mTc-MAA	18F-FDG
Alternative imaging	First-pass perfusion MRI		LGE MRI		4D flow MRI	∅
Radiation dose	+++	++	+++	++	+	++++

*18F-FDG, 18F-fluorodeoxyglucose; MAA, macroaggregated albumin; MRI, magnetic resonance imaging; N13-NH3, ammonia-N13; LGE, late gadolinium enhancement; PET, positron emission tomography; Rb-82, rubidium-82; SPECT, single-photon emission computed tomography; Tl-201, thallium-201; Tc-99m, technetium-99m.*

## Data Availability Statement

The original contributions presented in the study are included in the article/supplementary material, further inquiries can be directed to the corresponding author.

## Author Contributions

MV made substantial contributions to the conception and design of the work, drafted the work, and substantively revised it. MF, LM, JB, ZJ, and GT drafted the work. OV made substantial contributions to the conception and design of the work, has drafted the work, and substantively revised it. All authors have approved the submitted version and have agreed both to be personally accountable for the author’s own contributions and to ensure that questions related to the accuracy or integrity of any part of the work, even ones in which the author was not personally involved, are appropriately investigated, resolved, and the resolution documented in the literature.

## Conflict of Interest

The authors declare that the research was conducted in the absence of any commercial or financial relationships that could be construed as a potential conflict of interest. The reviewer LS declared a shared affiliation with the authors MV, LM, JB, and OV to the handling editor at the time of review.

## Publisher’s Note

All claims expressed in this article are solely those of the authors and do not necessarily represent those of their affiliated organizations, or those of the publisher, the editors and the reviewers. Any product that may be evaluated in this article, or claim that may be made by its manufacturer, is not guaranteed or endorsed by the publisher.

## References

[1] Mertens L Friedberg MK. The gold standard for noninvasive imaging in congenital heart disease: echocardiography. *Curr Opin Cardiol.* (2009) 24:119–24. 10.1097/HCO.0b013e328323d86f 19225295

[2] Alghamdi MH Ismail MI Yelbuz TM. Do we need more than a transthoracic echocardiography when evaluating children with congenital heart disease before cardiac surgery?. *Congenit Heart Dis.* (2016) 11:262–9. 10.1111/chd.12312 26560082

[3] Gerrah R Bardo DME Reed RD Sunstrom RE Langley SM. Adjustment of the surgical plan in repair of congenital heart disease: the power of cross-sectional imaging and three-dimensional visualization. *Congenit Heart Dis.* (2014) 9:E31–6. 10.1111/chd.12062 23601962

[4] Geiger J Callaghan FM Burkhardt BEU Valsangiacomo Buechel ER Kellenberger CJ. Additional value and new insights by four-dimensional flow magnetic resonance imaging in congenital heart disease: application in neonates and young children. *Pediatr Radiol.* (2021) 51:1503–17. 10.1007/s00247-020-04885-w 33313980 PMC8266722

[5] Kumar P Bhatia M. Role of computed tomography in pre- and postoperative evaluation of a double-outlet right ventricle. *J Cardiovasc Imaging.* (2021) 29:205–27. 10.4250/jcvi.2020.0196 34080329 PMC8318812

[6] Vallabhajosula S. *Molecular Imaging: Radiopharmaceuticals for PET and SPECT.* Berlin: Springer (2009). p. 372.

[7] Zanzonico P. Principles of nuclear medicine imaging: planar, SPECT, PET, multi-modality, and autoradiography systems. *Radiat Res.* (2012) 177:349–64. 10.1667/rr2577.1 22364319

[8] Werner RA Thackeray JT Diekmann J Weiberg D Bauersachs J Bengel FM. The changing face of nuclear cardiology: guiding cardiovascular care toward molecular medicine. *J Nucl Med.* (2020) 61:951–61. 10.2967/jnumed.119.240440 32303601 PMC7383072

[9] Villemain O Baranger J Jalal Z Lam C Calais J Pernot M Non-invasive imaging techniques to assess myocardial perfusion. *Expert Rev Med Devices.* (2020) 17:1133–44. 10.1080/17434440.2020.1834844 33044100

[10] Garcia EV. Physical attributes, limitations, and future potential for PET and SPECT. *J Nucl Cardiol.* (2012) 19:19–29. 10.1007/s12350-011-9488-3 22160631

[11] Erlandsson K Buvat I Pretorius PH Thomas BA Hutton BF. A review of partial volume correction techniques for emission tomography and their applications in neurology, cardiology and oncology. *Phys Med Biol.* (2012) 57:R119–59. 10.1088/0031-9155/57/21/R119 23073343

[12] Bailey DL. Transmission scanning in emission tomography. *Eur J Nucl Med.* (1998) 25:774–87. 10.1007/s002590050282 9662601

[13] Parker JA Coleman RE Grady E Royal HD Siegel BA Stabin MG SNM practice guideline for lung scintigraphy 4.0. *J Nucl Med Technol.* (2012) 40:57–65. 10.2967/jnmt.111.101386 22282651

[14] Srinivasan S Crandall JP Gajwani P Sgouros G Mena E Lodge MA Human radiation dosimetry for orally and intravenously administered 18F-FDG. *J Nucl Med.* (2020) 61:613–9. 10.2967/jnumed.119.233288 31628217 PMC9374043

[15] Vermeulen K Vandamme M Bormans G Cleeren F. Design and challenges of radiopharmaceuticals. *Semin Nucl Med.* (2019) 49:339–56. 10.1053/j.semnuclmed.2019.07.001 31470930

[16] Nunn AD. The cost of bringing a radiopharmaceutical to the patient’s bedside. *J Nucl Med.* (2007) 48:169. 17268008

[17] Knapp FF Mirzadeh S. The continuing important role of radionuclide generator systems for nuclear medicine. *Eur J Nucl Med.* (1994) 21:1151–65. 10.1007/BF00181073 7828627

[18] Cutler CS Bailey E Kumar V Schwarz SW Bom HS Hatazawa J Global issues of radiopharmaceutical access and availability: a nuclear medicine global initiative project. *J Nucl Med.* (2021) 62:422–30. 10.2967/jnumed.120.247197 32646881 PMC8049350

[19] Chhatriwalla AK Prieto LR Brunken RC Cerqueira MD Younoszai A Jaber WA. Preliminary data on the diagnostic accuracy of rubidium-82 cardiac PET perfusion imaging for the evaluation of ischemia in a pediatric population. *Pediatr Cardiol.* (2008) 29:732–8. 10.1007/s00246-008-9232-1 18458995

[20] Baggish AL Boucher CA. Radiopharmaceutical agents for myocardial perfusion imaging. *Circulation.* (2008) 118:1668–74. 10.1161/CIRCULATIONAHA.108.778860 18852377

[21] Nishiyama M Doi S Matsumoto A Nishioka M Hosokawa S Sasaki A Exercise-induced myocardial ischemia in a case of anomalous origin of the left main coronary artery from the noncoronary sinus of valsalva. *Pediatr Cardiol.* (2011) 32:1028–31. 10.1007/s00246-011-0051-4 21779965

[22] Slomka P Berman DS Alexanderson E Germano G. The role of PET quantification in cardiovascular imaging. *Clin Transl Imaging.* (2014) 2:343–58. 10.1007/s40336-014-0070-2 26247005 PMC4523308

[23] Schindler TH Quercioli A Valenta I Ambrosio G Wahl RL Dilsizian V. Quantitative assessment of myocardial blood flow – clinical and research applications. *Semin Nucl Med.* (2014) 44:274–93. 10.1053/j.semnuclmed.2014.04.002 24948151

[24] Cheezum MK Liberthson RR Shah NR Villines TC O’Gara PT Landzberg MJ Anomalous aortic origin of a coronary artery from the inappropriate sinus of valsalva. *J Am Coll Cardiol.* (2017) 69:1592–608. 10.1016/j.jacc.2017.01.031 28335843

[25] Van Hare GF Ackerman MJ Evangelista JAK Kovacs RJ Myerburg RJ Shafer KM Eligibility and disqualification recommendations for competitive athletes with cardiovascular abnormalities: task force 4: congenital heart disease: a scientific statement from the American heart association and American college of cardiology. *Circulation.* (2015) 132:e281–91. 10.1161/CIR.0000000000000240 26621645

[26] Frommelt P Lopez L Dimas VV Eidem B Han BK Ko HH Recommendations for multimodality assessment of congenital coronary anomalies: a guide from the American society of echocardiography: developed in collaboration with the society for cardiovascular angiography and interventions, Japanese society of echocar. *J Am Soc Echocardiogr.* (2020) 33:259–94. 10.1016/j.echo.2019.10.011 32143778

[27] Doan TT Molossi S Sachdeva S Wilkinson JC Loar RW Weigand JD Dobutamine stress cardiac MRI is safe and feasible in pediatric patients with anomalous aortic origin of a coronary artery (AAOCA). *Int J Cardiol.* (2021) 334:42–8. 10.1016/j.ijcard.2021.04.031 33892043

[28] Chatterjee A Watts TE Mauchley DC Iskandrian AE Law MA. Multimodality imaging of rare adult presentation of ALCAPA treated with takeuchi repair. *JACC Cardiovasc Interv.* (2018) 11:98–9. 10.1016/j.jcin.2017.09.016 29248407

[29] Seguchi M Nakanishi T Nakazawa M Doi S Momma K Takao A Myocardial perfusion after aortic implantation for anomalous origin of the left coronary artery from the pulmonary artery. *Eur Heart J.* (1990) 11:213–8. 10.1093/oxfordjournals.eurheartj.a059686 2318224

[30] Poryo M Khreish F Schäfers HJ Abdul-Khaliq H. A case of myocardial bridging as a rare cause of chest pain in children. *Clin Res Cardiol.* (2016) 105:279–81. 10.1007/s00392-015-0915-3 26349785

[31] Ergul Y Nisli K Kayserili H Karaman B Basaran S Dursun M Evaluation of coronary artery abnormalities in williams syndrome patients using myocardial perfusion scintigraphy and CT angiography. *Cardiol J.* (2012) 19:301–8. 10.5603/cj.2012.0053 22641550

[32] Lee ML. Regression of cardiac enzyme and ventriculocoronary communication in an infant with pulmonary atresia and intact ventricular septum after radiofrequency valvulotomy and valvuloplasty. *Pediatr Cardiol.* (2005) 26:792–6. 10.1007/s00246-005-0932-5 16082571

[33] Schulz R Heush G. Hibernating myocardium. *Heart.* (2013) 84:587–94. 10.1136/heart.84.6.587 11083733 PMC1729531

[34] Raja SG Shauq A Kaarne M. Outcomes after arterial switch operation for simple transposition. *Asian Cardiovasc Thorac Ann.* (2005) 13:190–8. 10.1177/021849230501300222 15905355

[35] van Wijk SWH van der Stelt F ter Heide H Schoof PH Doevendans PAFM Meijboom FJ. Sudden death due to coronary artery lesions long-term after the arterial switch operation: a systematic review. *Can J Cardiol.* (2017) 33:1180–7. 10.1016/j.cjca.2017.02.017 28778688

[36] Fricke TA Bell D Daley M d’Udekem Y Brizard CP Alphonso N The influence of coronary artery anatomy on mortality after the arterial switch operation. *J Thorac Cardiovasc Surg* (2020) 160:191–9.e1. 10.1016/j.jtcvs.2019.11.146 32222408

[37] Bonnet D Bonhoeffer P Piéchaud JF Aggoun Y Sidi D Planché C Long-term fate of the coronary arteries after the arterial switch operation in newborns with transposition of the great arteries. *Heart.* (1996) 76:274–9. 10.1136/hrt.76.3.274 8868989 PMC484520

[38] Legendre A Losay J Touchot-Koné A Serraf A Belli E Piot JD Coronary events after arterial switch operation for transposition of the great arteries. *Circulation.* (2003) 108:186–91. 10.1161/01.cir.0000087902.67220.2b 12970230

[39] Sterrett LE Schamberger MS Ebenroth ES Siddiqui AR Hurwitz RA. Myocardial perfusion and exercise capacity 12 years after arterial switch surgery for D-transposition of the great arteries. *Pediatr Cardiol.* (2011) 32:785–91. 10.1007/s00246-011-9975-y 21479909

[40] Yates RWM Marsden PK Badawi RD Cronin BF Anderson DR Tynan MJ Evaluation of myocardial perfusion using positron emission tomography in infants following a neonatal arterial switch operation. *Pediatr Cardiol.* (2000) 21:111–8. 10.1007/s002469910015 10754077

[41] Hauser M Bengel FM Kühn A Sauer U Zylla S Braun SL Myocardial blood flow and flow reserve after coronary reimplantation in patients after arterial switch and Ross operation. *Circulation.* (2001) 103:1875–80. 10.1161/01.cir.103.14.1875 11294806

[42] Sugiyama H Tsuda E Ohuchi H Yamada O Shiraishi I. Chronological changes in stenosis of translocated coronary arteries on angiography after the arterial switch operation in children with transposition of the great arteries: comparison of myocardial scintigraphy and angiographic findings. *Cardiol Young.* (2016) 26:638–43. 10.1017/S104795111500075X 25994511

[43] Tsuda T Baffa JM Octavio J Robinson BW Radtke W Mody T Identifying subclinical coronary abnormalities and silent myocardial ischemia after arterial switch operation. *Pediatr Cardiol.* (2019) 40:901–8. 10.1007/s00246-019-02085-4 30852629

[44] Rickers C Sasse K Buchert R Stern H Van Den Hoff J Lübeck M Myocardial viability assessed by positron emission tomography in infants and children after the arterial switch operation and suspected infarction. *J Am Coll Cardiol.* (2000) 36:1676–83. 10.1016/s0735-1097(00)00891-3 11079676

[45] Pizzi MN Franquet E Aguadé-Bruix S Manso B Casaldáliga J Cuberas-Borrós G Long-term follow-up assessment after the arterial switch operation for correction of dextro-transposition of the great arteries by means of exercise myocardial perfusion-gated SPECT. *Pediatr Cardiol.* (2014) 35:197–207. 10.1007/s00246-013-0759-4 23843104

[46] McCrindle BW Rowley AH Newburger JW Burns JC Bolger AF Gewitz M Diagnosis, treatment, and long-term management of Kawasaki disease: a scientific statement for health professionals from the American heart association. *Circulation.* (2017) 135:927–99. 10.1161/CIR.0000000000000484 28356445

[47] Senzaki H. Long-term outcome of Kawasaki disease. *Circulation.* (2008) 118:2763–72. 10.1161/CIRCULATIONAHA.107.749515 19106401

[48] Tsuda E Kamiya T Ono Y Kimura K Kurosaki K Echigo S. Incidence of stenotic lesions predicted by acute phase changes in coronary arterial diameter during Kawasaki disease. *Pediatr Cardiol.* (2005) 26:73–9. 10.1007/s00246-004-0698-1 15136903

[49] Kondo C Nakanishi T Sonobe T Tatara K Momma K Kusakabe K. Scintigraphic monitoring of coronary artery occlusion due to Kawasaki disease. *Am J Cardiol.* (1993) 71:681–5. 10.1016/0002-9149(93)91010-F 8447265

[50] Fukuda T Ishibashi M Shinohara T Miyake T Kudoh T Saga T. Follow-up assessment of the collateral circulation in patients with Kawasaki disease who underwent dipyridamole stress technetium-99m tetrofosmin scintigraphy. *Pediatr Cardiol.* (2005) 26:558–64. 10.1007/s00246-004-0726-1 16378208

[51] Fukazawa R Kobayashi J Ayusawa M Hamada H Miura M Mitani Y JCS/JSCS 2020 guideline on diagnosis and management of cardiovascular sequelae in Kawasaki disease. *Circ J.* (2020) 84:1348–407. 10.1253/circj.CJ-19-1094 32641591

[52] Karasawa K Ayusawa M Noto N Sumitomo N Okada T Harada K. Optimum protocol of technetium-99m tetrofosmin myocardial perfusion imaging for the detection of coronary stenosis lesions in Kawasaki disease. *J Cardiol.* (1997) 30:331–9. 9436075

[53] Fukuda T Ishibashi M Yokoyama T Otaki M Shinohara T Nakamura Y Myocardial ischemia in Kawasaki disease: evaluation with dipyridamole stress technetium 99m tetrofosim scintigraphy. *J Nucl Cardiol.* (2002) 9:632–7. 10.1067/mnc.2002.125915 12466788

[54] Fukushige J Takahashi N Ueda K Hijii T Igarashi H Ohshima A. Long-term outcome of coronary abnormalities in patients after Kawasaki disease. *Pediatr Cardiol.* (1996) 17:71–6. 10.1007/BF02505086 8833489

[55] Gravel H Curnier D Dallaire F Fournier A Portman M Dahdah N. Cardiovascular response to exercise testing in children and adolescents late after Kawasaki disease according to coronary condition upon onset. *Pediatr Cardiol.* (2015) 36:1458–64. 10.1007/s00246-015-1186-5 25951815

[56] Zanon G Zucchetta P Varnier M Vittadello F Milanesi O Zulian F. Do Kawasaki disease patients without coronary artery abnormalities need a long-term follow-up? A myocardial single-photon emission computed tomography pilot study. *J Paediatr Child Health.* (2009) 45:419–24. 10.1111/j.1440-1754.2009.01531.x 19712178

[57] Kashyap R Mittal BR Bhattacharya A Manojkumar R Singh S. Exercise myocardial perfusion imaging to evaluate inducible ischaemia in children with Kawasaki disease. *Nucl Med Commun.* (2011) 32:137–41. 10.1097/MNM.0b013e3283411c67 21127446

[58] Hauser M Bengel F Kuehn A Nekolla S Kaemmerer H Schwaiger M Myocardial blood flow and coronary flow reserve in children with “Normal” epicardial coronary arteries after the onset of Kawasaki disease assessed by positron emission tomography. *Pediatr Cardiol.* (2004) 25:108–12. 10.1007/s00246-003-0472-9 14668960

[59] Maron MS Olivotto I Maron BJ Prasad SK Cecchi F Udelson JE The case for myocardial ischemia in hypertrophic cardiomyopathy. *J Am Coll Cardiol.* (2009) 54:866–75. 10.1016/j.jacc.2009.04.072 19695469

[60] Haley JH Miller TD. Myocardial ischemia on thallium scintigraphy in hypertrophic cardiomyopathy: predictor of sudden cardiac death. *Circulation.* (2001) 104:E71–71. 10.1161/hc3801.096179 11571257

[61] Cecchi F Olivotto I Gistri R Lorenzoni R Chiriatti G Camici PG. Coronary microvascular dysfunction and prognosis in hypertrophic cardiomyopathy. *N Engl J Med.* (2003) 349:1027–35. 10.1056/NEJMoa025050 12968086

[62] Ziolkowska L Boruc A Sobielarska-Lysiak D Grzyb A Petryka-Mazurkiewicz J Mazurkiewicz Ł Prognostic significance of myocardial ischemia detected by single-photon emission computed tomography in children with hypertrophic cardiomyopathy. *Pediatr Cardiol.* (2021) 42:960–8. 10.1007/s00246-021-02570-9 33687492 PMC8110494

[63] Harris KM Spirito P Maron MS Zenovich AG Formisano F Lesser JR Prevalence, clinical profile, and significance of left ventricular remodeling in the end-stage phase of hypertrophic cardiomyopathy. *Circulation.* (2006) 114:216–25. 10.1161/CIRCULATIONAHA.105.583500 16831987

[64] Aguiar Rosa S Rocha Lopes L Fiarresga A Ferreira RC Mota Carmo M. Coronary microvascular dysfunction in hypertrophic cardiomyopathy: pathophysiology, assessment, and clinical impact. *Microcirculation.* (2021) 28:e12656. 10.1111/micc.12656 32896949

[65] Ommen SR Mital S Burke MA Day SM Deswal A Elliott P 2020 AHA/ACC guideline for the diagnosis and treatment of patients with hypertrophic cardiomyopathy: a report of the American college of cardiology/American heart association joint committee on clinical practice guidelines. *J Am Coll Cardiol.* (2020) 76:e159–240. 33229116 10.1016/j.jacc.2020.08.045

[66] Hallioglu O Ceylan Gunay E Unal S Erdogan A Balci S Citirik D. Gated myocardial perfusion scintigraphy in children with sickle cell anemia: correlation with echocardiography. *Rev Esp Med Nucl.* (2011) 30:354–9. 10.1016/j.remn.2011.02.003 21458111

[67] Jaeggi E Bolens M Friedli B. Reversible second degree atrioventricular block after a severe sickle cell crisis. *Pediatr Cardiol.* (1998) 19:171–3. 10.1007/s002469900272 9565512

[68] Pahl E Naftel DC Kuhn MA Shaddy RE Morrow WR Canter CE The impact and outcome of transplant coronary artery disease in a pediatric population: a 9-year multi-institutional study. *J Hear Lung Transplant.* (2005) 24:645–51. 10.1016/j.healun.2004.03.021 15949722

[69] Kindel SJ Law YM Chin C Burch M Kirklin JK Naftel DC Improved detection of cardiac allograft vasculopathy: a multi-institutional analysis of functional parameters in pediatric heart transplant recipients. *J Am Coll Cardiol.* (2015) 66:547–57. 10.1016/j.jacc.2015.05.063 26227194

[70] Jeewa A Dreyer WJ Kearney DL Denfield SW. The presentation and diagnosis of coronary allograft vasculopathy in pediatric heart transplant recipients. *Congenit Heart Dis.* (2012) 7:302–11. 10.1111/j.1747-0803.2012.00656.x 22497818

[71] Maiers J Hurwitz R. Identification of coronary artery disease in the pediatric cardiac transplant patient. *Pediatr Cardiol.* (2008) 29:19–23. 10.1007/s00246-007-9038-6 17891435

[72] Grant FD Treves ST. Nuclear medicine and molecular imaging of the pediatric chest: current practical imaging assessment. *Radiol Clin North Am.* (2011) 49:1025–51. 10.1016/j.rcl.2011.06.012 21889020

[73] Milanesi O Stellin G Zucchetta P. Nuclear medicine in pediatric cardiology. *Semin Nucl Med.* (2017) 47:158–69. 10.1053/j.semnuclmed.2016.10.008 28237004

[74] Roman KS Kellenberger CJ Farooq S MacGowan CK Gilday DL Yoo SJ. Comparative imaging of differential pulmonary blood flow in patients with congenital heart disease: magnetic resonance imaging versus lung perfusion scintigraphy. *Pediatr Radiol.* (2005) 35:295–301. 10.1007/s00247-004-1344-z 15490145

[75] Fathala A. Quantitative lung perfusion scintigraphy in patients with congenital heart disease. *Heart Views.* (2010) 11:109. 10.4103/1995-705X.76802 21577378 PMC3089824

[76] Sakaguchi T Watanabe Y Hirose M Takei K Yasukochi S. A novel diagnostic approach for assessing pulmonary blood flow distribution using conventional X-ray angiography. *PLoS One.* (2021) 16:e0253565. 10.1371/journal.pone.0253565 34185820 PMC8241113

[77] Haroutunian L Neill C Wagner H. Radioisotope scanning of the lung in cyanotic congenital heart disease. *Am J Cardiol.* (1969) 23:387–95. 10.1016/0002-9149(69)90519-0 5777687

[78] Sánchez-Crespo A Rohdin M Carlsson C Bergström SE Larsson SA Jacobsson H A technique for lung ventilation-perfusion SPECT in neonates and infants. *Nucl Med Commun.* (2008) 29:173–7. 10.1097/MNM.0b013e3282f25905 18094640

[79] Sanchez-Crespo A. Lung ventilation/perfusion single photon emission computed tomography (SPECT) in infants and children with nonembolic chronic pulmonary disorders. *Semin Nucl Med.* (2019) 49:37–46. 10.1053/j.semnuclmed.2018.10.006 30545515

[80] Masutani S Senzaki H Taketazu M Kobayashi J Kobayashi T Asano H Usefulness of selective contrast echocardiography and selective scintigraphy for the evaluation of pulmonary arteriovenous fistula in a patient with systemic arterial supply to a normal lung. *J Pediatr Surg.* (2005) 40:51–4. 10.1016/j.jpedsurg.2004.11.007 15793716

[81] Inuzuka R Aotsuka H Nakajima H Yamazawa H Sugamoto K Tatebe S Quantification of collateral aortopulmonary flow in patients subsequent to construction of bidirectional cavopulmonary shunts. *Cardiol Young.* (2008) 18:485–93. 10.1017/S104795110800259X 18634715

[82] Kim SJ Bae EJ Lee JY Lim HG Lee C Lee CH. Inclusion of hepatic venous drainage in patients with pulmonary Arteriovenous fistulas. *Ann Thorac Surg.* (2009) 87:548–53. 10.1016/j.athoracsur.2008.10.024 19161777

[83] Feltes TF Bacha E Beekman RH Cheatham JP Feinstein JA Gomes AS Indications for cardiac catheterization and intervention in pediatric cardiac disease: a scientific statement from the American Heart Association. *Circulation.* (2011) 123:2607–52. 10.1161/CIR.0b013e31821b1f10 21536996

[84] Latus H Kuehne T Beerbaum P Apitz C Hansmann G Muthurangu V Cardiac MR and CT imaging in children with suspected or confirmed pulmonary hypertension/pulmonary hypertensive vascular disease. Expert consensus statement on the diagnosis and treatment of paediatric pulmonary hypertension. The European paediatric pulmonary vascular disease network, endorsed by ISHLT and DGPK. *Heart.* (2016) 102:ii30–5. 27053695 10.1136/heartjnl-2015-308246

[85] Spencer R Valencia Villeda G Takeda K Rosenzweig EB. Chronic thromboembolic pulmonary hypertension in a child with sickle cell disease. *Front Pediatr.* (2020) 8:363. 10.3389/fped.2020.00363 32850520 PMC7396518

[86] Drubach LA Jenkins KJ Stamoulis C Palmer EL Lee EY. Evaluation of primary pulmonary vein stenosis in children: comparison of radionuclide perfusion lung scan and angiography. *Am J Roentgenol.* (2015) 205:873–7. 10.2214/AJR.14.13581 26397339

[87] Sridharan S Derrick G Deanfield J Taylor AM. Assessment of differential branch pulmonary blood flow: a comparative study of phase contrast magnetic resonance imaging and radionuclide lung perfusion imaging. *Heart.* (2006) 92:963–8. 16775104 10.1136/hrt.2005.071746 PMC1860721

[88] Boothroyd A McDonald E Carty H. Lung perfusion scintigraphy in patients with congenital heart disease: sensitivity and important pitfalls. *Nucl Med Commun.* (1996) 17:33–9. 10.1097/00006231-199601000-00007 8692471

[89] Morgan CT Mertens L Grotenhuis H Yoo SJ Seed M Grosse-Wortmann L. Understanding the mechanism for branch pulmonary artery stenosis after the arterial switch operation for transposition of the great arteries. *Eur Heart J Cardiovasc Imaging.* (2017) 18:180–5. 10.1093/ehjci/jew046 27025515

[90] Harisankar CNB Mittal BR Agrawal KL Abrar ML Bhattacharya A. Utility of high fat and low carbohydrate diet in suppressing myocardial FDG uptake. *J Nucl Cardiol.* (2011) 18:926–36. 21732228 10.1007/s12350-011-9422-8

[91] Jadvar H Alavi A Mavi A Shulkin BL. PET in pediatric diseases. *Radiol Clin North Am.* (2005) 43:135–52. 15693653 10.1016/j.rcl.2004.09.008

[92] Fernández-Hidalgo N Tornos Mas P. Epidemiology of infective endocarditis in spain in the last 20 years. *Rev Española Cardiol.* (2013) 66:728–33. 10.1016/j.rec.2013.05.002 24773679

[93] Saby L Laas O Habib G Cammilleri S Mancini J Tessonnier L Positron emission tomography/computed tomography for diagnosis of prosthetic valve endocarditis: increased valvular 18F- fluorodeoxyglucose uptake as a novel major criterion. *J Am Coll Cardiol.* (2013) 61:2374–82. 23583251 10.1016/j.jacc.2013.01.092

[94] Dixon G Christov G. Infective endocarditis in children: an update. *Curr Opin Infect Dis.* (2017) 30:257–67. 28319472 10.1097/QCO.0000000000000370

[95] Ly R Compain F Gaye B Pontnau F Bouchard M Mainardi J-L Predictive factors of death associated with infective endocarditis in adult patients with congenital heart disease. *Eur Hear J Acute Cardiovasc Care.* (2020). [Epub ahead of print]. 10.1177/2048872620901394 31990202

[96] Habib G Lancellotti P Antunes M Bongiorni MG Casalta J-P Del Zotti F 2015 ESC guidelines for the management of infective endocarditis the task force for the management of infective endocarditis of the European society of cardiology (ESC) Endorsed by?: European association for cardio-thoracic surgery. *Eur Heart J.* (2015) 36:3075–123. 10.1093/eurheartj/ehv319 26320109

[97] Nishimura RA Otto CM Bonow RO Carabello BA Erwin JP Fleisher LA 2017 AHA/ACC focused update of the 2014 AHA/ACC guideline for the management of patients with valvular heart disease: a report of the American college of cardiology/American heart association task force on clinical practice guidelines. *Circulation.* (2017) 135:1159–95. 10.1161/CIR.0000000000000503 28298458

[98] Gomes A Glaudemans AWJM Touw DJ van Melle JP Willems TP Maass AH Diagnostic value of imaging in infective endocarditis: a systematic review. *Lancet Infect Dis.* (2016) 17:e1–14. 10.1016/S1473-3099(16)30141-4 27746163

[99] Venet M Jalal Z Ly R Malekzadeh-Milani S Hascoët S Fournier E Diagnostic value of 18F-fluorodeoxyglucose positron emission tomography computed tomography in prosthetic pulmonary valve infective endocarditis. *JACC Cardiovasc Imaging.* (2021) 15:299–308. 34538632 10.1016/j.jcmg.2021.07.015

[100] Dell’Aquila AM Mastrobuoni S Alles S Wenning C Henryk W Schneider SRB Contributory role of fluorine 18-fluorodeoxyglucose positron emission tomography/computed tomography in the diagnosis and clinical management of infections in patients supported with a continuous-flow left ventricular assist device. *Ann Thorac Surg.* (2016) 101:87–94. 10.1016/j.athoracsur.2015.06.066 26433521

[101] Pizzi MN Dos-Subirà L Roque A Fernández-Hidalgo N Cuéllar-Calabria H Pijuan Domènech A 18F-FDG-PET/CT angiography in the diagnosis of infective endocarditis and cardiac device infection in adult patients with congenital heart disease and prosthetic material. *Int J Cardiol.* (2017) 248:396–402. 10.1016/j.ijcard.2017.08.008 28807509

[102] Sarrazin JF Philippon F Tessier M Guimond J Molin F Champagne J Usefulness of fluorine-18 positron emission tomography/computed tomography for identification of cardiovascular implantable electronic device infections. *J Am Coll Cardiol.* (2012) 59:1616–25. 10.1016/j.jacc.2011.11.059 22538331

[103] Amraoui S Tlili G Sohal M Berte B Hindié E Ritter P Contribution of PET imaging to the diagnosis of septic embolism in patients with pacing lead endocarditis. *JACC Cardiovasc Imaging.* (2016) 9:283–90. 10.1016/j.jcmg.2015.09.014 26897683

[104] de Vaugelade C Mesguich C Nubret K Camou F Greib C Dournes G Infections in patients using ventricular-assist devices: comparison of the diagnostic performance of 18 F-FDG PET/CT scan and leucocyte-labeled scintigraphy. *J Nucl Cardiol.* (2019) 26:42–55. 10.1007/s12350-018-1323-7 29948892

[105] Kawamura J Ueno K Taimura E Matsuba T Imoto Y Jinguji M Case report: 18F-FDG PET-CT for diagnosing prosthetic device-related infection in an infant with CHD. *Front Pediatr.* (2021) 9:584741. 10.3389/fped.2021.584741 33763393 PMC7982821

[106] Bos D De Wolf D Cools B Eyskens B Hubrechts J Boshoff D Infective endocarditis in patients after percutaneous pulmonary valve implantation with the stent-mounted bovine jugular vein valve: clinical experience and evaluation of the modified Duke criteria. *Int J Cardiol.* (2021) 323:40–6. 10.1016/j.ijcard.2020.08.058 32860844

[107] Pizzi MN Roque A Fernández-Hidalgo N Cuéllar-Calabria H Ferreira-González I Gonzàlez-Alujas MT Improving the diagnosis of infective endocarditis in prosthetic valves and intracardiac devices with 18F-fluordeoxyglucose positron emission tomography/computed tomography angiography: initial results at an infective endocarditis referral center. *Circulation.* (2015) 132:1113–26. 10.1161/CIRCULATIONAHA.115.015316 26276890

[108] Chawla SC Federman N Zhang D Nagata K Nuthakki S McNitt-Gray M Estimated cumulative radiation dose from PET/CT in children with malignancies: a 5-year retrospective review. *Pediatr Radiol.* (2010) 40:681–6. 10.1007/s00247-009-1434-z 19967534 PMC2847163

[109] López-Mora DA Carrió I Flotats A. Digital PET vs analog PET: clinical implications?. *Semin Nucl Med.* (2021) 52:302–11. 10.1053/j.semnuclmed.2021.10.004 34836617

[110] Erba PA Conti U Lazzeri E Sollini M Doria R De Tommasi SM Added value of 99mTc-HMPAO-labeled leukocyte SPECT/ CT in the characterization and management of patients with infectious endocarditis. *J Nucl Med.* (2012) 53:1235–43. 10.2967/jnumed.111.099424 22787109

[111] Sarrazin J-F Philippon F Trottier M Tessier M. Role of radionuclide imaging for diagnosis of device and prosthetic valve infections. *World J Cardiol.* (2016) 8:534. 10.4330/wjc.v8.i9.534 PMC5039355 27721936

[112] Gratz S Behr T Herrmann A Meller J Conrad M Zappel H Immunoscintigraphy (BW 250/183) in neonates and infants with fever of unknown origin. *Nucl Med Commun.* (1998) 19:1037–45. 10.1097/00006231-199811000-00003 9861620

[113] Aydin F Cengiz A Gugor F. Tc-99m labeled HMPAO white blood cell scintigraphy in pediatric patients. *Mol Imaging Radionucl Ther.* (2012) 21:13–8. 23487346 10.4274/Mirt.165 PMC3590957

[114] Datz FL Seabold JE Brown ML Forstrom LA Greenspan BS McAfee JG Procedure guideline for technetium-99m-HMPAO-labeled leukocyte scintigraphy for suspected infection/inflammation. *J Nucl Med.* (1997) 38:987–90. 9189157

[115] Agius C Rakotonirina H Lacoeuille F Bouchet F Vervueren L Le Jeune JJ Infection de prothèse vasculaire: 18TEP-FDG vs scintigraphie aux leucocytes marqués (planaires et TEMP/TDM). *Med Nucl.* (2011) 35:628–40. 10.1016/j.mednuc.2011.09.004

[116] Burke A Virmani R. Pediatric heart tumors. *Cardiovasc Pathol.* (2008) 17:193–8. 18402818 10.1016/j.carpath.2007.08.008

[117] Beroukhim RS Prakash A Valsangiacomo Buechel ER Cava JR Dorfman AL Festa P Characterization of cardiac tumors in children by cardiovascular magnetic resonance imaging: a multicenter experience. *J Am Coll Cardiol.* (2011) 58:1044–54. 10.1016/j.jacc.2011.05.027 21867841

[118] Tyebally S Chen D Bhattacharyya S Mughrabi A Hussain Z Manisty C Cardiac tumors: JACC cardiooncology state-of-the-art review. *JACC CardioOncol.* (2020) 2:293–311. 34396236 10.1016/j.jaccao.2020.05.009 PMC8352246

[119] Snoussi NEH Radi F Oussou Y Manouri K El Hattab FZ Cherti M. Left ventricular metastasis of osteosarcoma: a report of an unusual case. *J Card Surg.* (2020) 35:3596–9. 32939815 10.1111/jocs.15021

[120] Coccia P Ruggiero A Rufini V Maurizi P Attinà G Marano R Cardiac metastases of Ewing sarcoma detected by 18F-FDG PET/CT. *J Pediatr Hematol Oncol.* (2012) 34:236–8. 10.1097/MPH.0b013e318242754d 22395217

[121] Strauss HW Zaret BL Hurley PJ Natarajan TK Pitt B. A scintiphotographic method for measuring left ventricular ejection fraction in man without cardiac catheterization. *Am J Cardiol.* (1971) 28:575–80. 10.1016/0002-9149(71)90100-7 5116974

[122] Schaffer MS De Souza M Olley PM Rowe R Gilday D. Qualitative phase analysis in pediatric nuclear cardiology: isolation of cardiac chambers and identification of asynchronous contraction patterns. *Pediatr Cardiol.* (1984) 5:179–84. 10.1007/BF02427042 6099554

[123] Schaffer MS Samuels LD Nouri S. Equilibrium radionuclide ventriculography in single ventricle. *Clin Nucl Med.* (1985) 10:699–702. 4075653 10.1097/00003072-198510000-00007

[124] Oguz D Olgunturk R Gucuyener K Acikgoz G Tunaoglu FA. Comparison between MUGA and echocardiography in patients with muscular dystrophy in the early detection of cardiac involvement. *Pediatr Cardiol.* (1998) 19:150–4. 10.1007/s002469900264 9565507

[125] Dae MW. Pediatric nuclear cardiology. *Semin Nucl Med.* (2007) 37:382–90. 17707243 10.1053/j.semnuclmed.2007.05.003

[126] Maltz DL Treves S. Quantitative radionuclide angiocardiography: determination of Qp:Qs in children. *Circulation.* (1973) 47:1049–56. 4705571 10.1161/01.cir.47.5.1049

[127] Baker EJ Ellam SV Lorber A Jones OD Tynan MJ Maisey MN. Superiority of radionuclide over oximetric measurement of left to right shunts. *Br Heart J.* (1985) 53:535–40. 10.1136/hrt.53.5.535 3994867 PMC481805

[128] Malčić I Senečić I Težak S Ivančević D Kniewald H. Radioangioscintigraphy and Doppler echocardiography in the quantification of left-to-right shunt. *Pediatr Cardiol.* (2000) 21:240–3. 10.1007/s002460010049 10818183

[129] Kuehn A Vogt M Schwaiger M Ewert P Hauser M. Ventricular sympathetic innervation in patients with transposition of the great arteries after arterial switch operation and rastelli procedure: impact of arterial dissection and coronary reimplantation. *Circ J.* (2014) 78:1717–22. 10.1253/circj.cj-13-1594 24882547

[130] Possner M Buechel RR Vontobel J Mikulicic F Gräni C Benz DC Myocardial blood flow and cardiac sympathetic innervation in young adults late after arterial switch operation for transposition of the great arteries. *Int J Cardiol.* (2020) 299:110–5. 10.1016/j.ijcard.2019.07.041 31337551

[131] Nygaard S Christensen AH Rolid K Nytrøen K Gullestad L Fiane A Autonomic cardiovascular control changes in recent heart transplant recipients lead to physiological limitations in response to orthostatic challenge and isometric exercise. *Eur J Appl Physiol.* (2019) 119:2225–36. 10.1007/s00421-019-04207-5 31407088 PMC6763412

[132] Kelesidis I Travin MI. Use of cardiac radionuclide imaging to identify patients at risk for arrhythmic sudden cardiac death. *J Nucl Cardiol.* (2012) 19:142–52. 22130965 10.1007/s12350-011-9482-9

[133] Ohuchi H Negishi J Miyake A Sakaguchi H Miyazaki A Yamada O. Long-term prognostic value of cardiac autonomic nervous activity in postoperative patients with congenital heart disease. *Int J Cardiol.* (2011) 151:296–302. 10.1016/j.ijcard.2010.05.062 20580104

[134] Ono S Ohuchi H Miyazaki A Abe T Kiso K Yamada O. Heterogeneity of ventricular sympathetic nervous activity is associated with clinically relevant ventricular arrhythmia in postoperative patients with tetralogy of fallot. *Pediatr Cardiol.* (2015) 36:1515–22. 10.1007/s00246-015-1195-4 25981565

[135] Müller KD Jakob H Neuzner J Grebe SF Schlepper M Pitschner HF. 123I-metaiodobenzylguanidine scintigraphy in the detection of irregular regional sympathetic innervation in long QT syndrome. *Eur Heart J.* (1993) 14:316–25. 10.1093/eurheartj/14.3.316 8458350

[136] Olguntürk R Turan L Tunaoglu FS Kula S Gökçora N Karabacak NI. Abnormality of the left ventricular sympathetic nervous function assessed by I-123 metaiodobenzylguanidine imaging in pediatric patients with neurocardiogenic syncope. *Pacing Clin Electrophysiol.* (2003) 26:1926–30. 10.1046/j.1460-9592.2003.00297.x 14516330

[137] Kutanzi KR Lumen A Koturbash I Miousse IR. Pediatric exposures to ionizing radiation: carcinogenic considerations. *Int J Environ Res Public Health.* (2016) 13:1057. 10.3390/ijerph13111057 PMC5129267 27801855

[138] Andreassi MG Picano E. Reduction of radiation to children our responsibility to change. *Circulation.* (2014) 130:135–7. 24914036 10.1161/CIRCULATIONAHA.114.010699

[139] Velasco-Sanchez D Lambert R Turpin S Laforge S Fournier A Lapierre C Right ventricle myocardial perfusion scintigraphy: feasibility and expected values in children. *Pediatr Cardiol.* (2012) 33:295–301. 10.1007/s00246-011-0128-0 21968577

[140] Fahey FH Treves ST Adelstein SJ. Minimizing and communicating radiation risk in pediatric nuclear medicine. *J Nucl Med Technol.* (2012) 40:13–24. 22393223 10.2967/jnumed.109.069609

[141] Lassmann M Biassoni L Monsieurs M Franzius C. The new EANM paediatric dosage card: additional notes with respect to F-18. *Eur J Nucl Med Mol Imaging.* (2008) 35:1666–8. 10.1007/s00259-008-0799-9 18574583

[142] Gelfand MJ Parisi MT Treves ST. Pediatric radiopharmaceutical administered doses: 2010 North American consensus guidelines. *J Nucl Med.* (2011) 52:318–22. 21233182 10.2967/jnumed.110.084327

[143] Society of nuclear medicine and molecular imaging website. *SNMMI Procedures Standards.* (2014). Available online at: http://www.snmmi.org/ClinicalPractice/content.aspx?ItemNumber=6414&navItemNumber=10790 (accessed March 2022).

[144] Cho SG Kim J Song HC. Radiation safety in nuclear medicine procedures. *Nucl Med Mol Imaging.* (2017) 51:11–6. 28250853 10.1007/s13139-016-0406-0 PMC5313457

[145] Dickson J Eberlein U Lassmann M. The effect of modern PET technology and techniques on the EANM paediatric dosage card. *Eur J Nucl Med Mol Imaging.* (2021) 49:1964–9. 10.1007/s00259-021-05635-2 34910233 PMC9016049

[146] Xie T Lee C Bolch WE Zaidi H. Assessment of radiation dose in nuclear cardiovascular imaging using realistic computational models. *Med Phys.* (2015) 42:2955–66. 10.1118/1.4921364 26127049 PMC5148206

[147] Magill D Alavi A. Radiation safety concerns related to PET/computed tomography imaging for assessing pediatric diseases and disorders. *PET Clin.* (2020) 15:293–8. 10.1016/j.cpet.2020.03.012 32498985

[148] Queiroz MA De Galiza Barbosa F Buchpiguel CA Cerri GG. Positron emission tomography/magnetic resonance imaging (PET/MRI): an update and initial experience at HC-FMUSP. *Rev Assoc Med Bras.* (2018) 64:71–84. 10.1590/1806-9282.64.01.71 29561945

[149] Theruvath AJ Ilivitzki A Muehe A Theruvath J Gulaka P Kim C A PET / MR imaging approach for the integrated assessment of chemotherapy-induced brain, heart, and bone injuries in pediatric cancer survivors: a pilot study. *Radiology.* (2018) 285:971–9. 10.1148/radiol.2017170073 28777701 PMC5708284

[150] Villemain O Malekzadeh-Milani S Sitefane F Mostefa-Kara M Boudjemline Y. Radiation exposure in transcatheter patent ductus arteriosus closure: time to tune?. *Cardiol Young.* (2018) 28:653–60. 29347998 10.1017/S1047951117002839

